# Leukotriene Receptor Antagonist, Montelukast Ameliorates L-NAME-Induced Pre-eclampsia in Rats through Suppressing the IL-6/Jak2/STAT3 Signaling Pathway

**DOI:** 10.3390/ph15080914

**Published:** 2022-07-24

**Authors:** Walaa Yehia Abdelzaher, Gomaa Mostafa-Hedeab, Haitham Ahmed Bahaa, Ahmad Mahran, Michael Atef Fawzy, Sara Mohamed Naguib Abdel Hafez, Nermeen N. Welson, Remon Roshdy Rofaeil

**Affiliations:** 1 Department of Pharmacology, Faculty of Medicine, Minia University, Minia 61511, Egypt; walaayehia22@yahoo.com (W.Y.A.); roshdyremon@yahoo.com (R.R.R.); 2 Pharmacology Department, Medical College, Jouf University, Sakaka 11564, Saudi Arabia; 3 Pharmacology Department, Faculty of Medicine, Beni-Suef University, Beni Suef 62511, Egypt; 4 Department of Obstetrics and Gynecology, Faculty of Medicine, Minia University, Minia 61511, Egypt; haitham_bahaa@yahoo.com (H.A.B.); ezzeldin_ahmad@yahoo.com (A.M.); 5 Department of Biochemistry, Faculty of Pharmacy, Minia University, Minia 61511, Egypt; michaelfawzy777@yahoo.com; 6 Department of Histology and Cell Biology, Faculty of Medicine, Minia University, Minia 61511, Egypt; sara_histology@yahoo.com; 7 Department of Forensic Medicine and Clinical Toxicology, Faculty of Medicine, Beni-Suef University, Beni Suef 62511, Egypt; nermeennemr@yahoo.com; 8 Department of Pharmacology, Deraya University, New Minia 61111, Egypt

**Keywords:** leukotriene receptor antagonist, L-NAME, pre-eclampsia, IL-6/Jak2/STAT3

## Abstract

Aims: To investigate the potential protective role of montelukast (Mont) in the pre-eclampsia rat model induced by L-NG-Nitro arginine methyl ester (L-NAME). Methods and materials: Thirty-two pregnant female albino Wistar rats were assigned to four groups: the control group: pregnant rats received vehicles; the Mont group: pregnant rats received Mont (10 mg/kg/day, p.o.) from the 6th to the 18th day of gestation; the L-NAME group: pregnant rats received L-NAME (50 mg/kg/day, i.p.) from the 9th to the 18th day of gestation; the Mont/L-NAME group: pregnant rats received Mont (10 mg/kg/day, p.o.) from the 6th to the 18th day of gestation and L-NAME (50 mg/kg/day, i.p.) from the 9th to the 18th day of gestation. Placental, hepatic, and renal malondialdehyde (MDA), total nitrites (NOx), interleukin 6 (IL-6), and tumor necrosis factor (TNF)-α were determined. Serum alanine transaminase (ALT), aspartate transaminase (AST), creatinine, urea, 24-h urinary protein, and the placental growth factor (PGF) were measured. Histopathological examinations of the placental, hepatic, and renal tissues were also performed. In addition, placental, hepatic, and renal Janus kinase 2 (Jak2) and signal transducer and activator of transcription 3 (STAT3) immunoblotting were performed. Key findings: Mont improves oxidative stress, IL-6, TNF-α, ALT, AST, creatinine, urea, 24-h urinary protein, PGF, Jak2, and STAT3 which were all affected by L-NAME. Moreover, the histopathological assessment indicated that Mont restored the normal architecture that was markedly disturbed by L-NAME. Significance: Mont exerted the biochemical and histopathological amelioration of L-NAME-caused pre-eclampsia through its anti-inflammatory, anti-oxidant function and suppression of the IL-6/Jak2/STAT3 signaling pathway.

## 1. Introduction

The major cause of perinatal morbidity and mortality is pre-eclampsia (PE), affecting about 5–7% of pregnant women and developing after 20 weeks of gestation, resulting in maternal and fetal deaths worldwide [1]. PE is characterized by hypertension, edema, and proteinuria with a high risk of maternal death, fetal death, premature labor, and intrauterine fetal growth retardation [1,2,3].

Unfortunately, the definite etiology of PE is unclear and without effective treatments to date. It is primarily a complex disorder in which the etiology is not due to a single cause, such as genetic, immunogenic, or environmental variables but rather to a complicated combination of numerous factors. PE can cause stroke, renal failure, pulmonary edema, liver rupture, and eclampsia if left untreated [4]. Multi-organ damage has been reported with PE, which is related to increased reactive oxygen species generation, the induction of inflammation, and acceleration of apoptosis [5].

It is well known that PE is linked to placental development disruption, which is accompanied by cellular, molecular, immunological, and vascular changes. Furthermore, abnormal placentation and shallow trophoblast invasion within the uterus result in incomplete spiral artery remodeling, which may lead to placental hypoxia, vascular endothelial dysfunction, inhibition of placental formation, and trophoblast immaturity. Failed decidual differentiation prior to pregnancy has been linked to impaired trophoblast invasion and its consequences [6,7].

Moreover, the only effective treatment for PE is delivery, which results in a newborn with intrauterine growth retardation and premature birth as well as various neonatal sequelae and elevated fetal pro-inflammatory markers [8]. Despite the high risk of maternal and fetal complications, not all cases of PE suffer from these complications. It was reported that the incidence of HELLP syndrome was 9.1%. The incidence of maternal mortality was 1.7%, and 49.6% of the babies were lost through stillbirths and early neonatal deaths [9].

Nitric oxide (NO) plays an important role in the regulation of the cardiovascular system during pregnancy. L-NG-Nitro arginine methyl ester (L-NAME), a known inhibitor of NO synthesis, can cause PE. When given to animals throughout the middle-to-late stages of pregnancy, it causes a state that mimics pre-eclampsia in humans, characterized by decreased placental perfusion, increased reactive oxygen species (ROS) generation, hypertension, and proteinuria [10].

Montelukast (Mont) is one of the most frequently used drugs in chronic asthmatic females planning for pregnancy and is safely used during pregnancy without increasing the risk of congenital anomalies [11]. It is a selective cysteinyl leukotriene receptor antagonist, with anti-oxidant, anti-inflammatory, and anti-apoptotic effects in different models of organ damage [12,13,14].

It was reported that montelukast produced peripheral analgesia through the activation of the L-arginine/NO pathway and that L-NAME antagonized its action [15].

Therefore, the current study was performed to investigate the protective effect of Mont in PE in rats. For this purpose, various biochemical, histological, and immunohistochemical approaches were sought to unravel the potential underlying mechanisms of action. 

## 2. Results

### 2.1. Effect of Mont on MAP in a Pre-eclampsia Model in Rats

**Figure 1:**
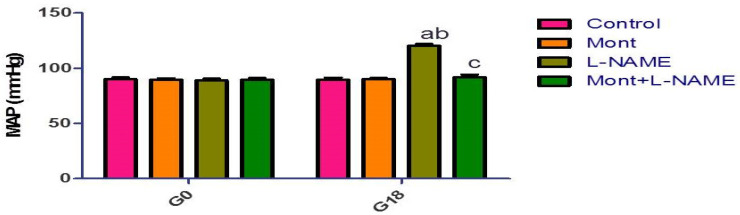
Effect of montelukast on blood pressure in a pre-eclampsia model in rats. rats/group). (a) Significant (*p* < 0.05) difference from the control group. (b) Significant (*p* < 0.05) difference from the Mont group. (c) Significant (*p* < 0.05) difference from the L-NAME group. [Mont: montelukast; L-NAME: L-NG-Nitro arginine methyl ester].

There was no significant change in MAP between the groups on day 0. Meanwhile, on day 18, there was a significant increase in MAP in the L-NAME group compared to the control and Mont groups. The Mont + L-NAME group showed significantly decreased MAP compared to the L-NAME group (Figure 1). 

### 2.2. Effect of Mont on 24 h Urine Protein in a Pre-eclampsia Model in Rats

**Figure 2:**
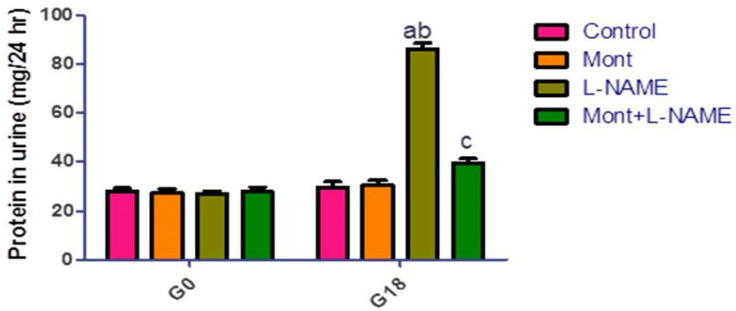
Effect of montelukast on 24 h urine protein in a pre-eclampsia model in rats. rats/group). (a) Significant (*p* < 0.05) difference from the control group. (b) Significant (*p* < 0.05) difference from the Mont group. (c) Significant (*p* < 0.05) difference from the L-NAME group. [Mont: montelukast; L-NAME: L-NG-Nitro arginine methyl ester].

There was no significant change in the 24 h urine protein between the groups on day 0. Meanwhile, on day 18, there was a significant increase in 24 h urine protein in the L-NAME group compared to the control and Mont groups. The Mont + L-NAME group showed significantly decreased 24 h urine protein compared to the L-NAME group (Figure 2). 

### 2.3. Effect of Mont on Placental Parameters in a Pre-eclampsia Model in Rats

In Table 1, the L-NAME group showed a significant decrease in PGF levels together with an increase in MDA, NOx, IL-6, and TNF-α in placental tissue compared to the control and Mont groups. On the other hand, Mont + L-NAME showed significantly increased PGF levels with a significant reduction in MDA, NOx, IL-6, and TNF-α in placental tissue compared to the L-NAME group.

**Figure 3:**
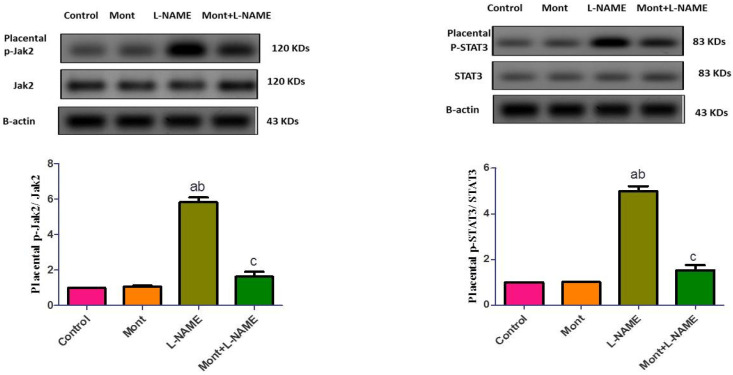
Effect of montelukast on placental p-Jak2 and p-STAT3 in a pre-eclampsia model in rats. The results show the mean ± SEM (8 rats/group). (a) Significant (*p* < 0.05) difference from the control group. (b) Significant (*p* < 0.05) difference from the Mont group. (c) Significant (*p* < 0.05) difference from the L-NAME group. [Mont: montelukast; L-NAME: L-NG-Nitro arginine methyl ester; Jak2: Janus kinase 2; STAT3: signal transducer and activator of transcription 3].

In Figure 3, placental p-Jak2 and p-STAT3 expressions were elevated in the L-NAME group compared to the control and Mont groups. In contrast, the Mont + L-NAME group showed significantly declined placental p-Jak2 and p-STAT3 expressions in comparison to the L-NAME group. 

### 2.4. Effect of Mont on Hepatic Parameters in a Pre-eclampsia Model in Rats

In Table 2, L-NAME showed significantly increased serum ALT and AST compared to the control and Mont groups. Similarly, in hepatic tissue, it showed increased MDA, NOx, IL-6, and TNF-α. In contrast, in the Mont + L-NAME group, a significant reduction in ALT, AST, MDA, NOx, IL-6, and TNF-α was noticed compared to the L-NAME group.

**Figure 4:**
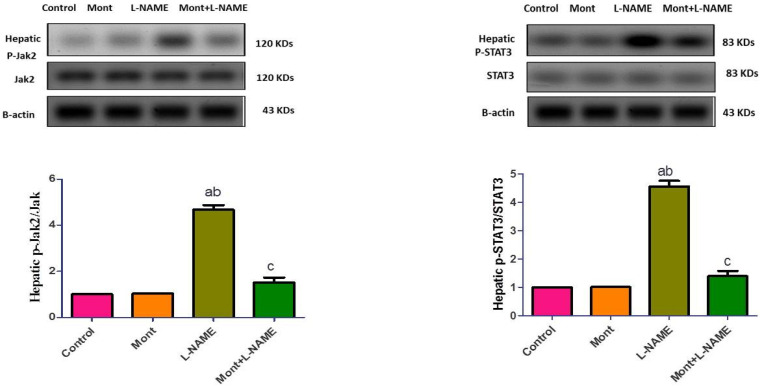
Effect of montelukast on hepatic p-Jak2 and p-STAT3 in a pre-eclampsia model in rats. The results show the mean ± SEM (8 rats/group). (a) Significant (*p* < 0.05) difference from the control group. (b) Significant (*p* < 0.05) difference from the Mont group. (c) Significant (*p* < 0.05) difference from the L-NAME group. [Mont: montelukast; L-NAME: L-NG-Nitro arginine methyl ester; Jak2: Janus kinase 2; STAT3: signal transducer and activator of transcription 3].

Compared to the control and Mont groups, hepatic p-Jak2 and p-STAT3 expressions were elevated in the L-NAME group. On the other hand, the Mont + L-NAME group showed significantly suppressed hepatic p-Jak2 and p-STAT3 expressions compared to the L-NAME group (Figure 4). 

### 2.5. Effect of Mont on Renal Parameters in a Pre-eclampsia Model in Rats

In Table 3, the L-NAME group showed a significant elevation in serum urea, serum creatinine, renal MDA, renal NOx, renal IL-6, and renal TNF-α compared to the control and Mont groups. In contrast, the Mont + L-NAME group showed significantly decreased urea, creatinine, MDA, NOx, IL-6, and TNF-α compared to the L-NAME group.

**Figure 5:**
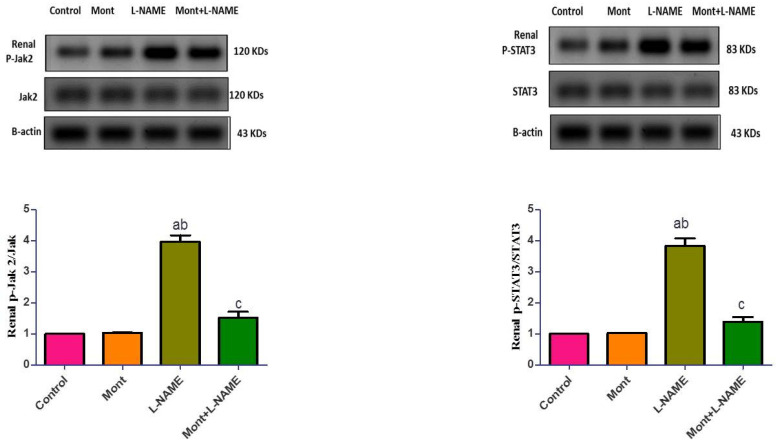
Effect of montelukast on renal p-Jak2 and p-STAT3 in a pre-eclampsia model in rats. The results show the mean ± SEM (8 rats/group). (a) Significant (*p* < 0.05) difference from the control group. (b) Significant (*p* < 0.05) difference from the Mont group. (c) Significant (*p* < 0.05) difference from the L-NAME group. [Mont: montelukast; L-NAME: L-NG-Nitro arginine methyl ester; Jak2: Janus kinase 2; STAT3: signal transducer and activator of transcription 3].

In Figure 5, renal p-Jak2 and p-STAT3 expressions were elevated in the L-NAME group compared to the control and Mont groups. In contrast, the Mont + L-NAME group showed significantly suppressed renal p-Jak2 and p-STAT3 expressions in comparison to the L-NAME group. 

### 2.6. Histopathological Results

**Figure 6:**
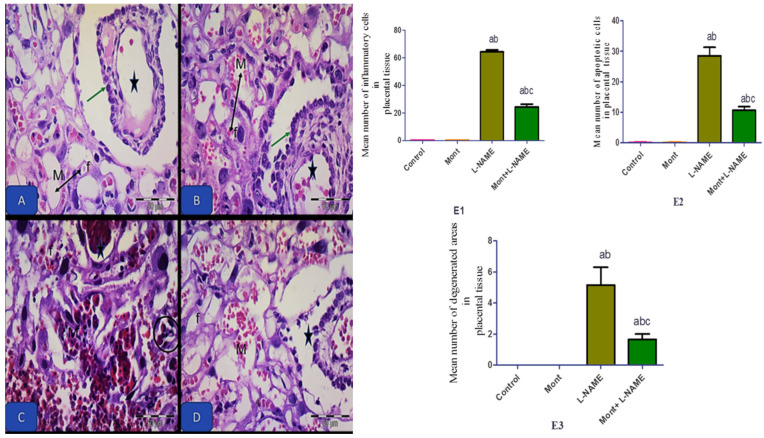
Effect of montelukast on the placenta in a pre-eclampsia model in rats. Photomicrographs of the control (**A**) and the Mont (**B**) groups showing full-term rat placenta at the level of labyrinth displaying normal interramal membrane and its constituents (double-headed arrow) with normal fetal capillaries (f) and normal maternal sinus (M). Notice the chorionic projection with their layers (green arrow) and inner fetal blood capillary with normal RBCs (star). (**C**) The L-NAME group showing distorted chorionic projection (star). Notice the dilated, congested maternal (M) vessels and fetal capillaries (f) and inflammatory cell infiltration (circle). (**D**) The Mont + L-NAME group showing amelioration of all previously mentioned pathological changes. Notice the apparent normal chorionic villi (star), maternal (M) vessels, and fetal capillaries (f). H&E × 400. (**E1**–**E3**) The histopathological changes in renal tissue in all groups. The data are mean ± SEM (8 rats/group). (a) Significant (*p* < 0.05) difference from the control group. (b) Significant (*p* < 0.05) difference from the Mont group. (c) Significant (*p* < 0.05) difference from the L-NAME group. [Mont: montelukast; L-NAME: L-NG-Nitro arginine methyl ester]. H&E × 400, scale bare = 50 µm.

Photomicrographs of the control and Mont groups showed full-term rat placenta at the level of labyrinth displaying normal interramal membrane and its constituents with normal fetal capillaries and normal maternal sinus. The L-NAME group showed distorted chorionic projection, dilated and congested maternal vessels, and fetal capillaries with inflammatory cell infiltration. The Mont + L-NAME group showed amelioration of all previously mentioned pathological changes (Figure 6).

**Figure 7:**
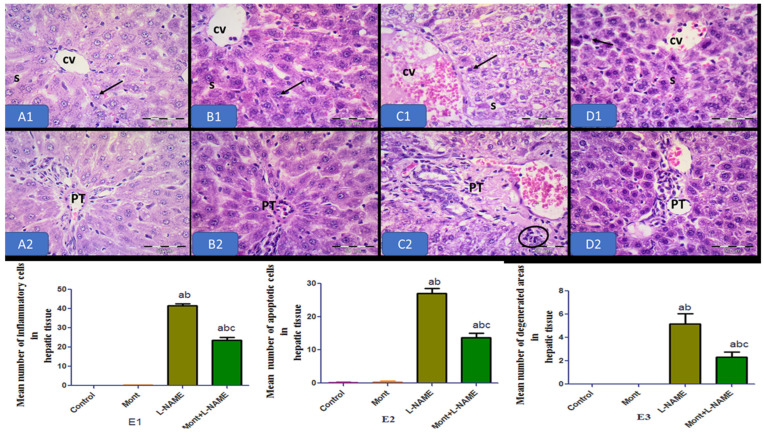
Effect of montelukast on the liver in a pre-eclampsia model in rats. Photomicrographs of liver tissue of the control (**A1**,**A2**) and the Mont (**B1**,**B2**) groups showing the normal organization of the liver tissue. Notice the hepatocytes (arrows) with vesicular nuclei radiating from the central veins (CV) and separated by blood sinusoids (S). Notice the portal tract (PT). (**C1**,**C2**) The L-NAME group showing dilated, congested central veins (CV) and portal tracts (PT). Notice the more numerous apoptotic cells (arrows) and inflammatory cell infiltration (circle). (**D1**,**D2**) The Mont + L-NAME group showing more or less normal liver tissue. Notice the normal central veins (CV), portal tract (PT), and hepatocytes (arrows). Notice the less numerous apoptotic cells (arrows) H&E × 400. (**E1**–**E3**) The histopathological changes in renal tissue in all groups. The data are mean ± SEM (8 rats/group). (a) Significant (*p* < 0.05) difference from the control group. (b) Significant (*p* < 0.05) difference from the Mont group. (c) Significant (*p* < 0.05) difference from the L-NAME group. [Mont: montelukast; L-NAME: L-NG-Nitro arginine methyl ester.] H&E × 400, scale bare = 50 µm.

Hepatic photomicrographs of the control and Mont groups showed normal organization of the hepatic tissue. The hepatocytes showed vesicular nuclei radiating from the central veins and separated by blood sinusoids in normal architecture. The L-NAME group showed dilated, congested central veins and portal tracts, with more numerous apoptotic cells and inflammatory cell infiltration. The Mont + L-NAME group showed more or less normal hepatic tissue (Figure 7).

**Figure 8:**
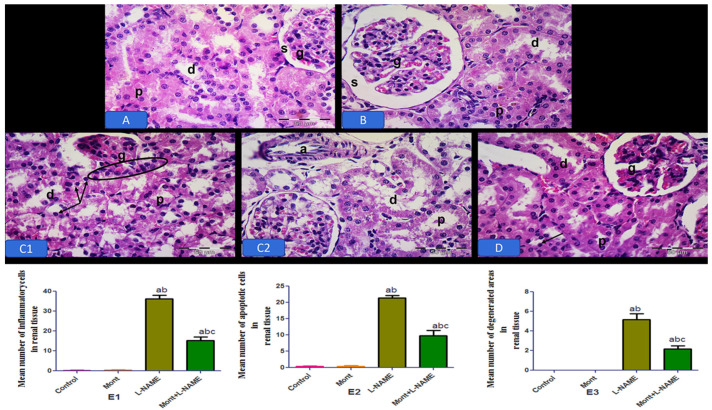
Effect of montelukast on kidneys in a pre-eclampsia model in rats. Photomicrographs of the renal cortex of the control (**A**) and the Mont (**B**) groups displaying normal organization of the renal cortex. Notice the glomerular capillary tufts (g) and Bowman’s space (s). PCTs (p) have a narrow lumen and a highly acidophilic cytoplasm. DCTs (d) have a wider lumen and less acidophilic cytoplasm with vesicular nuclei (arrow). (**C1**,**C2**) The L-NAME group showing disturbed lobular architecture with dilated interlobular arteries (a). Notice the atrophic glomerulus (g), dilated PCTs (p), DCTs (d), and the numerous apoptotic cells (arrows) lining the tubules. Inflammatory cell infiltration is also detected (circle). (**D**) The Mont + L-NAME group showing the restoration of normal architecture. The renal tissue approaches the normal structure. H&E × 400. (**E1**–**E3**) The histopathological changes in renal tissue in all groups. The data are mean ± SEM (8 rats/group). (a) Significant (*p* < 0.05) difference from the control group. (b) Significant (*p* < 0.05) difference from the Mont group. (c) Significant (*p* < 0.05) difference from the L-NAME group. [Mont: montelukast; L-NAME: L-NG-Nitro arginine methyl ester.] H&E × 400, scale bare = 50 µm.

Renal photomicrographs of the control and Mont groups showed normal organization of the renal cortex, the glomerular capillary tufts and Bowman’s space. Proximal convoluted tubules (PCTs) have a narrow lumen and a highly acidophilic cytoplasm. Distal convoluted tubules (DCTs) have a wider lumen and less acidophilic cytoplasm with vesicular nuclei. The L-NAME group showed disturbed lobular architecture with dilated interlobular arteries with atrophic glomerulus, dilated PCTs (P), DCTs, and numerous apoptotic cells lining the tubules. Inflammatory cell infiltration was also detected. The Mont + L-NAME group showed the restoration of normal architecture (Figure 8). The histopathological changes in placental, hepatic, and renal tissue in all groups are shown in histograms in Figure 6, Figure 7 and Figure 8, respectively.

## 3. Discussion

PE, which is a multi-systemic disorder, is one of the most life-threatening pregnancy-related hypertensive disturbances. Despite the severity of PE, its precise pathogenesis is not yet fully understood and is currently an area of active research.

In the current work, L-NAME, a nitric oxide synthase (NOS) inhibitor, was used to induce a PE rat model, which is well proven to be able to imitate PE-like manifestations including hypertension and proteinuria [10]. Due to the antagonizing actions of Mont on inflammation, apoptosis, and oxidative stress, it has the potential to play a key role in the prevention of PE-related consequences, including hepatic and renal problems [4].

In accordance with previous studies [16,17,18], which evaluated the effects of estradiol, paeoniflorin, and vagal stimulation, respectively, in the L-NAME model for the induction of PE, our study reveals that L-NAME caused elevated blood pressure and urinary protein excretion. It also induced placental toxicity, evidenced by the reduction in PGF and the induction of inflammatory status shown by the increase in IL-6 and TNF-α. Furthermore, it induced oxidative stress shown by elevated MDA and NOx levels. Similarly, the main causes of PE, including endothelial dysfunction [18], excessive inflammation [19], immunological disturbance, and oxidative stress [2,5] were well-defined. 

L-NAME also increased serum urea and creatinine concentrations as well as increased MDA, NOx, IL-6, and TNF-α in renal tissue. This deterioration in renal function and the increase in oxidative stress and inflammatory parameters were associated with histopathological evidence of kidney affection (disturbed lobular architecture, dilated interlobular arteries, atrophic glomerulus, numerous apoptotic cells lining the tubules, and inflammatory cell infiltration). Earlier studies proved these findings. Shu et al. used different doses for the induction of PE [20]. In addition, Zuo and his colleague who used melatonin confirmed these changes with L-NAME [21].

Similarly, L-NAME increased serum ALT and AST, hepatic MDA, hepatic NOx, hepatic IL-6, and hepatic TNF-α. The increments in indicators of liver damage, oxidative stress, and inflammatory parameters were associated with changes in the histopathological picture of the liver (dilated, congested central veins and portal tracts, with numerous apoptotic cells and inflammatory cell infiltration). Previous reports mentioned similar biochemical and histopathological changes in the liver of pre-eclamptic rats injected with L-NAME that caused intense mononuclear inflammatory infiltrate and thickening of the muscle tunica of arterial vessels, mainly in the periportal area [22,23].

The question is: how is PE associated with renal and hepatic injury? The pathway through which these injuries occur is still under intense debate. However, it is established that PE arises, in part, from inappropriate placentation and trophoblast invasion, causing placental ischemia and encouraging the disproportionate production of anti-angiogenic (sFlt-1) and pro-angiogenic (PLGF and VEGF) agents. This imbalance activates inflammatory cells, causing them to release auto-antibodies and inflammatory cytokines as well as causing oxidative stress, all of which contribute to generalized maternal endothelial dysfunction in various organ beds, resulting in hypertension, endothelins, blood coagulation, and organ damage [24].

However, in the L-NAME-induced pre-eclamptic rat model, treatment with Mont rescued the elevated blood pressure and proteinuria, reduced serum urea and creatinine concentrations, AST, ALT, renal and hepatic inflammatory and oxidative stress parameters, and attenuated kidney and liver histopathological changes. These findings suggest that Mont ameliorated the nephritic and hepatic dysfunctions caused by L-NAME. Many studies have reported that Mont has nephroprotective and hepatoprotective effects by reducing oxidative stress and inflammation in other models of tissue injury, such as sepsis-induced kidney injury, cisplatin-induced nephrotoxicity, lipopolysaccharide-induced hepatotoxicity, and cecal ligation and puncture-induced sepsis [12,13,25,26,27].

The Jak/STAT signaling pathway has an undeniable role in cell growth, differentiation, and survival. This pathway is also involved in the inflammatory response and immune function. The Jak family includes Jak1, 2, 3, and TYK2, and the STAT protein family contains seven members: STAT1, 2, 3, 4, 5A, 5B, and 6 [28,29].

STAT3 depends on the IL-6/Jak2/STAT3 signaling pathway in its activity, which may explain why there are contradictory reports on the activity of STAT3 in many diseases such as tumors and gastrointestinal problems [30].

A pleiotropic cytokine, IL-6, is released in large amounts during infection, autoimmunity, tissue damage, and cancer. Low IL-6 levels may exert beneficial actions such as tissue regeneration, while the chronically elevated production of IL-6 may result in tissue injury [31].

The stimulation of the Jak/STAT signaling system is an important pathogenic mechanism that causes endothelial cell dysfunction [32,33]. Phosphorylation of Jak2 and STAT3 in human placenta and trophoblast cells has been identified in several previous investigations. In numerous studies, the activation of the Jak/STAT signaling pathway has been shown to have a crucial role in PE. The expression levels of Jak/STAT signaling pathway-associated proteins in rat placental tissues were found to be lower in normotensive pregnant rats compared to hypertensive pregnant rats. It was stated that the activation of this pathway is associated with the induction of inflammation and oxidative stress in many cells, such as placental trophoblasts, platelets, and vascular endothelial cells [34,35,36,37].

In agreement with the aforementioned studies, in the current study, L-NAME increased IL-6 levels, p-Jak2, and p-STAT3 expressions, which were accompanied by the induction of inflammation and oxidative stress in the placenta, liver, and kidney. These changes caused tissue damage, which manifested as hypertension, proteinuria, and worsening kidney and liver functions.

In contrast, Mont reduced IL-6 levels, p-Jak2, and p-STAT3 expressions, which were associated with the amelioration of inflammation and oxidative stress in the placenta, liver, and kidney. This protective effect was shown by the improvement in the histopathological picture of these tissues and confirmed by the reduction in blood pressure, the protein level in urine, and the restoration of kidney and liver functions.

Surprisingly, it was reported that JAK/STAT signaling regulated extracellular trophoblast growth and participated in PE development by affecting expression levels of phosphorylated STAT3 and STAT1. It was also reported that JAK/STAT3 signaling is indispensable for the regulation of the invasion of trophoblast [38,39]. Similarly, curcumin was reported to ameliorate inadequate trophoblast invasion and spiral artery remodeling, significant histopathological alterations observed in PE through inhibition of the JAK/STAT pathway [40,41]. Therefore, this study provides additional evidence that supports the activation of JAK/STAT signaling pathways in PE.

In conclusion, L-NAME induced increased blood pressure and proteinuria as well as placental, hepatic, and renal injuries. The administration of Mont alleviated all these changes through antagonizing the inflammatory status and oxidative stress, probably through IL-6/Jak2/STAT3 signaling inhibition. Accordingly, we suggest that the inhibition of Jak2/STAT3 signaling can enhance placental, hepatic, and renal functions, preventing PE and represents a novel therapeutic strategy for PE treatment.

## 4. Materials and Methods

### 4.1. Ethics

Animal handling, treatment, and authentication were conducted in accordance with the requirements of the Institutional Ethical Committee (Faculty of Medicine, Minia University, Egypt) for the care of experimental animals as well as the NIH Guide for the care and use of laboratory animals (Approval number, 8192021).

### 4.2. Animals and Experimental Design

The National Research Centre in Giza, Egypt, provided 18 adult male Sprague Dawley rats (12–14 weeks old, 200–250 g) and 36 adult female Sprague Dawley rats (12–15 weeks old, 200–250 g). The rats were housed in the animal house of the Department of Pharmacology at the Faculty of Medicine, Minia University. For 7 days before the start of the experiment, the rats were housed under regulated conditions of temperature (22 ± 2 °C) and humidity (50 ± 10%) with a 12/12 h reversed light/dark cycle and ad libitum access to a standard pellet diet and tap water.

Male rats were mated with all the female rats overnight. The day the spermatozoa were found in the vaginal smear was considered to be day 0 of pregnancy. The mating period lasted 15 days in total until a duplicate number of groups was achieved. Non-mated female rats during this time were considered infertile and were discarded from the study [42]. The total number of pregnant female rats was 32, with a mating success rate of 90%. Pregnant rats were housed individually under the aforementioned standard laboratory conditions.

Pregnant rats were randomized into four groups (8 rats in each group) as follows:

Control group: Pregnant rats received vehicles; distilled water i.p. and carboxymethyl cellulose p.o.

Mont group: pregnant rats were given Mont (10 mg/kg/day, p.o.) from the 6th to 18th day of gestation. Mont was dissolved in carboxymethyl cellulose [43].

L-NAME group: pregnant rats were given L-NAME dissolved in distilled water (50 mg/kg/day, i.p.) from the 9th to 18th day of gestation [16].

Mont/L-NAME group: pregnant rats were given Mont (10 mg/kg/day, p.o.) from the 6th to 18th day of gestation and L-NAME (50 mg/kg/day, i.p.) from the 9th to 18th day of gestation.

### 4.3. Collection of Samples and Measurements

The 24 h urine of rats was collected using metabolic cages on days 0 and 18 of pregnancy to determine urinary protein. A Rat Urinary Protein kit was used to determine the level of urine protein (Biodiagnostic, Egypt). Measurement of blood pressure (BP) was performed using the non-invasive tail-cuff method on days 0 and 18 after the rats were pre-warmed in a heating chamber at 37 °C for 15 min. The blood pressure was measured five times, and the average result for each rat was calculated.

At the end of our experiment, under light halothane anesthesia, each rat was anesthetized and then euthanized on day 19 of gestation. Blood was collected from the abdominal aorta and centrifuged at 3000× *g* for 15 min (JanetzkiT30 centrifuge, Germany), with sera kept at −80 °C for further biochemical analysis. Placenta, liver, and kidney were excised and washed. The tissue samples were divided into three parts: the first was stored at −80 °C until used, the second was embedded in paraffin for the histopathological examination, and the third was used for immunoblotting.

#### 4.3.1. Serum Measurements 

Alanine transaminase (ALT) and aspartate transaminase (AST) were measured using commercial kits purchased from Spectrum Diagnostic, Egypt.

Serum urea and creatinine were measured using kits purchased from Biomed, Egypt.

#### 4.3.2. Tissue Measurements 

To assess placental, hepatic, and renal oxidative stress biomarkers, lipid peroxidation was measured as a thiobarbituric acid-reacting substance and displayed as equivalents of malondialdehyde (MDA) using 1,1,3,3-tetra methoxy propane as a standard [44]. Total nitrite, the stable oxidation end product of nitric oxide (NOx), was estimated after the reduction in nitrate to nitrite by copperized cadmium. The concentration of nitric dioxide (NO2^−^) was based on the Griess reaction, the reaction of nitrite with a mixture of naphthyl ethylenediamine and sulfanilamide [45]. 

Tumor necrosis factor alpha (TNF-α) was measured using ELISA kits obtained from Elabscience Biotechnology Inc., Houston, TX, USA (Cat.No.: E-EL-R2856). Interleukin-6 (IL-6) was measured using ELISA kits obtained from Nanjing Jincheng Bioengineering, Nanjing, China (Cat. No: H007). Placental growth factor (PGF) was determined using ELISA kits obtained from MyBioSource, San Diego, CA, USA (Cat.No.: MBS268190). The evaluation was undertaken according to the manufacturer instructions. 

Placental, hepatic, and renal homogenates (50 μg of total proteins) were boiled for five minutes with 2-mercaptoethanol containing loading buffer and then, applied to 12% sodium dodecyl sulfate-polyacrylamide gel electrophoresis (SDS-PAGE) to do running for two hours at 100 V. After electrophoresis, proteins were applied to polyvinylidene fluoride (PVDF) membranes. Blocking for one hour was performed in a Tris-buffered saline (TBS-T) solution which contained 5% (*w*/*v*) non-fat milk and 0.05% Tween-20. Incubation with primary antibodies (1:1000) was completed overnight at 4 °C for: rabbit anti-Janus kinase 2 (JAK2) (# 3230S, Cell Signaling Technology, Danvers, MA, USA); rabbit anti-pY1007/1008 JAK2 (# 3776S, Cell Signaling Technology, Danvers, MA, USA); rabbit anti-signal transducer and activator of transcription 3 (STAT3) (# 12640, Cell Signaling Technology, Danvers, MA, USA); rabbit anti-pY705 STAT3 (# 9145S, Cell Signaling Technology, Danvers, MA, USA); and β-actin (Santa Cruz Biotechnology, Santa Cruz, CA, USA). Secondary antibodies of horseradish peroxidase-conjugated polyclonal goat anti-rabbit immunoglobulin were added at a dilution of 1:5000 in the blocking buffer. Chemiluminescence was used for the visualization of bands with the help of a chemiluminescence kit using a luminescent image analyzer (LAS- 4100, Fujifilm Co., Tokyo, Japan). Protein bands of different groups were quantified as fold change relative to the control group by applying Image J Software.

#### 4.3.3. Histological Examination

Placental, hepatic, and renal tissue sections (5μ) from all groups were stained with hematoxylin and eosin (H&E). All specimens were examined and photographed using a high-resolution color digital camera mounted on a BX51 microscope (Olympus, Tokyo, Japan) and connected to a computer programmed with LC micro application software in the light microscopic unit of the Histology Department, Faculty of Medicine, Minia University.

### 4.4. Statistical Analysis 

Results were displayed as the mean ± S.E.M. The one-way analysis of variance (ANOVA) was carried out and followed by Tukey’s test to analyze the data for statistically significant variance. GraphPad Prism software was used for statistical calculations (version 5.01 for Windows, GraphPad Software, San Diego, CA, USA (www.graphpad.com, accessed on 10 July 2022).

## 5. Conclusions

L-NAME induced increased blood pressure and proteinuria as well as placental, hepatic, and renal injuries. The administration of Mont alleviated all these changes through antagonizing the inflammatory status and oxidative stress, probably through IL-6/Jak2/STAT3 signaling inhibition. Accordingly, we suggest that the inhibition of Jak2/STAT3 signaling can enhance placental, hepatic, and renal functions, preventing PE and represents a novel therapeutic strategy for PE treatment.

## Figures and Tables

**Table 1:** Effect of montelukast on placental parameters in a pre-eclampsia model in rats.

Groups	PGF (pg/mg Tissue)	Placental MDA (nmol/g Tissue)	Placental NOx (nmol/g Tissue)	Placental IL-6 (pg/g Tissue)	Placental TNF-α (pg/g Tissue)
Control	1.96 ± 0.12	29.7 ± 2.72	159.6 ± 7.01	42.8 ± 2.47	22 ± 1.15
Mont	1.88 ± 0.09	30.18 ± 2.92	148.7 ± 6.69	44.8 ± 1.89	23.8 ± 1.11
L-NAME	0.70 ± 0.04 ^ab^	84.2 ± 3.39 ^ab^	256 ± 3.69 ^ab^	78.3 ± 2.41 ^ab^	69.8 ± 3.29 ^ab^
Mont + L-NAME	1.81 ± 0.07 ^c^	37.7 ± 2.57 ^c^	138.4 ± 3.75 ^c^	48.8 ± 2.59 ^c^	28.9 ± 1.46 ^c^

The results show the mean ± SEM (8 rats/group). (^a^) Significant (*p* < 0.05) difference from the control group. (^b^) Significant (*p* < 0.05) difference from the Mont group. (^c^) Significant (*p* < 0.05) difference from the L-NAME group. [Mont: montelukast; L-NAME: L-NG-Nitro arginine methyl ester; PGF: placental growth factor; MDA: malondialdehyde; NOx: total nitrite/nitrate; IL-6: interleukin 6; TNF-α: tumor necrosis factor alpha].

**Table 2:** Effect of montelukast on hepatic parameters in a pre-eclampsia model in rats.

Groups	Serum AST (U/L)	Serum ALT (U/L)	Hepatic MDA (nmol/g Tissue)	Hepatic NOx (nmol/g Tissue)	Hepatic IL-6 (pg/g Tissue)	Hepatic TNF-α (pg/g Tissue)
Control	34.2 ± 1.58	25.9 ± 1.25	54.6 ± 3.21	65.4 ± 3.57	55.9 ± 2.17	73.9 ± 2.66
Mont	36.6 ± 2.27	26.4 ± 0.75	55.7 ± 2.91	68.8 ± 2.97	54.5 ± 2.26	77.5 ± 3.22
L-NAME	82.9 ± 3.73 ^ab^	79.0 ± 2.78 ^ab^	95.7 ± 2.66 ^ab^	132 ± 3.06 ^ab^	101 ± 2.47 ^ab^	170 ± 0 4.97 ^ab^
Mont + L-NAME	39.4 ± 1.30 ^c^	30.2 ± 1.85 ^c^	57.3 ± 2.40 ^c^	71.2 ± 3.16 ^c^	57.8 ± 2.54 ^c^	81 ± 4.13 ^c^

The results show the mean ± SEM (8 rats/group). (^a^) Significant (*p* < 0.05) difference from the control group. (^b^) Significant (*p* < 0.05) difference from the Mont group. (^c^) Significant (*p* < 0.05) difference from the L-NAME group. [Mont: montelukast; L-NAME: L-NG-Nitro arginine methyl ester; AST: aspartate transaminase; ALT: alanine transaminase; MDA: malondialdehyde; NOx: total nitrite/nitrate; IL-6: interleukin 6; TNF-α: tumor necrosis factor alpha].

**Table 3:** Effect of montelukast on renal parameters in a pre-eclampsia model in rats.

Groups	Urea (mg/dL)	Serum Creatinine (mg/dL)	Renal MDA (nmol/gm Tissue)	Renal NOx (nmol/gm Tissue)	Renal IL-6 (pg/g Tissue)	Renal TNF-α (pg/g Tissue)
Control	40.6 ±1.13	0.96 ± 0.05	75.8 ± 3.15	50.6 ± 2.88	42.3 ± 2.03	39 ± 2.44
Mont	42.8 ± 1.52	0.99 ± 0.06	78.6 ± 3.72	50.1 ± 3.57	43.8 ± 2.86	41.8 ± 2.18
L-NAME	80.5 ± 2.77 ^ab^	3.39 ± 0.21 ^ab^	160 ± 2.85 ^ab^	96.8 ± 3.92 ^ab^	72.9 ± 2.74 ^ab^	97.3 ± 3.69 ^ab^
Mont + L-NAME	43.3 ±1.39 ^c^	1.03 ±0.08 ^c^	84 ± 2.86 ^c^	56.5 ± 2.27 ^c^	47.7 ± 1.96 ^c^	48.1 ± 2.23 ^c^

The results show the mean ± SEM (8 rats/group). (^a^) Significant (*p* < 0.05) difference from the control group. (^b^) Significant (*p* < 0.05) difference from the Mont group. (^c^) Significant (*p* < 0.05) difference from the L-NAME group. [Mont: montelukast; L-NAME: L-NG-Nitro arginine methyl ester; MDA: malondialdehyde; NOx: total nitrite/nitrate; IL-6: interleukin 6; TNF-α: tumor necrosis factor alpha].

## Data Availability

The data presented in this study are available from the corresponding author upon reasonable request.
